# A Question of Nature: Some Antigens are Bound to be Allergens

**DOI:** 10.3389/fimmu.2014.00373

**Published:** 2014-08-05

**Authors:** Lain Pontes-de-Carvalho, José Mengel

**Affiliations:** ^1^Centro de Pesquisas Gonçalo Moniz, Fundação Oswaldo Cruz, Salvador, Brazil; ^2^Social Changes, Asthma and Allergy in Latin America – SCAALA – Program, Salvador, Brazil; ^3^Instituto Oswaldo Cruz, Fundação Oswaldo Cruz, Rio de Janeiro, Brazil; ^4^Faculty of Medicine of Petropolis, FMP-FASE, Petrópolis, Brazil

**Keywords:** allergy, helminth, evolution, Th2, allergen, cross-reactivity, allergenicity

Many allergens are proteins that have homologs in metazoan parasites, such as helminths [reviewed in Ref. (1)]. Indeed, it was proposed that this is a feature of most or even all environmental allergens (1). It has also been hypothesized that allergenicity itself would depend on the homology between allergen and helminth (2): a molecule would be an allergen because it would share antigenic determinants with a parasite – a question of nature. This notion was based on (i) the fact that the immunity against helminths is associated with a Th2, IgE-producing immune response (3–8); (ii) the fact that most allergic diseases are caused by this same type of immune response (9); (iii) the premise that there would be a propensity to mount Th2 immune responses against helminths and, of course, against antigens sharing antigenic determinants with them.

The sharing of antigenic determinants with parasites would not be the only feature of a molecule that would lead to the elicitation of a Th2 immune response. For instance, the honey bee venom phospholipase A2 elicits a Th2 immune response by disrupting the epithelial cell membrane and releasing IL-33, a cytokine that promotes the Th2 immune response (10). In addition, four (out of more than 20) aeroallergen groups from the dust mite *Dermatophagoides pteronyssinus* are proteases (11–13). They might elicit a Th2 immune response by directly inducing the release of another Th2 immune response-promoting cytokine, the IL-4, from mast cells, as suggested by an *in vitro* study (14) or by acting as described above for the bee venom phospholipase A2.

The allergenicity of a molecule, therefore, could depend on factors (13) other than the sharing of antigenic determinants with helminths. The hypothesis that allergenicity could depend on this sharing of antigenic determinants could, anyway, still hold for most allergens, which are not proteases. It does not entail, however, that someone would have first to be infected by a helminth for cross-reactive memory Th2 cells to be formed and later on become activated by an allergen. This would be contradicted by the increased prevalence of allergic reactions in populations in which helminth infections are reduced, such as those from industrialized countries (15, 16).

In order to account for the elicitation of cross-reactive Th2 immune responses by allergens sharing antigenic determinants with helminths in individuals who have not been in contact with those parasites, an expanded helminth-reactive Th2 cell population should constitutively exist in these individuals. The evolution of this expanded helminth-reactive lymphocyte population would have to had been accounted for, of course, by the effects of random mutation and natural selection. In addition, the selective pressure could not had varied to a great extent along relatively short periods of time, and in this case, immune responses against ubiquitous and more conserved antigenic molecules should have been the best candidates to be the targets of the selection.

However, natural selection cannot significantly modify the repertoire of the immune system as a whole, due to the enormous variability of the antigen-recognizing receptor that occurs during the somatic differentiation of the lymphocytes: one cannot select for a character that will appear after the selection. Therefore, the selection should be exerted on a subpopulation of lymphocytes with a relatively limited repertoire of antigen recognition. This subpopulation does indeed exist.

Non-template (N)-nucleotide additions in the DNA encoding the antigen-recognizing moyeties (complementarity-determining regions) of T-cell receptors/antibodies are the major determinants of immunological diversity (17, 18). These N-nucleotide additions do not occur (in the case of the mouse) or occur in a reduced degree (in the case of human beings) early in ontogeny (19). Cells with receptors containing proteins encoded by these genes without N-nucleotide additions (N^−^) persist in adult life with an antigen-recognition repertoire rather less variant than that of the cells whose genes coding the antigen receptors had the N-nucleotide additions (N^+^) (19). Newborn N^−^ T cells have been shown to be Th2 cells, and they, and their N^−^ B cell counterparts, are found in high frequencies in the mucosa-associated lymphoid tissues (20–23).

Because these cells are Th2 cells, are preferentially present in the mucosa, and have a reduced antigen-recognizing repertoire, and despite the fact that they, as a whole, have been shown to be multireactive (19), we propose that, due to natural selection, their N^−^ repertoire is biased to the recognition of ubiquitous parasite antigens. This hypothesis is amenable to experimental testing.

Through the mechanism proposed above, therefore, its possible that parasites – and even other antigens, as venoms and toxins (10, 24–26) – to which our ancestors, primates and non-primates, have been exposed for millions of years, have shaped our immune repertoire at the level of the germ line genes, so that the immune system would mount stronger responses against them (and against antigens sharing determinants with them) than against other antigens – a kind of “phylogenetic adaptative memory.”

The hypothesis described above proposes that both helminths and most allergens would stimulate the same basic Th2 repertoire. Helminth infections usually lead to the production of IgE antibodies characterized by low levels of somatic mutation and very little signs of antigenic selection (27). On the contrary, the IgE antibodies found in allergic populations display a high degree of somatic mutation and clear signs of antigenic selection (28). Therefore, one would have to speculate that, although helminth infections and allergens would stimulate cells with the same basic N^−^ repertoire, allergens would also recruit cells with the more sophisticated N^+^ repertoire, leading to the production of high-affinity IgE antibodies, whereas helminths would lead to the production of low-affinity, less mutated, multireactive N^–^ encoded IgE antibodies. The reason for that may be the enforcement of regulatory mechanisms triggered or reinforced by the helminth infection, such as the stimulation of regulatory Tr1 and/or Foxp-3^+^ T cells that could abort the recruitment of the N^+^ cells (29). In addition, the multireactive, low-affinity IgE produced during some helminth infections could even exert a regulatory effect on the allergic reaction, by competing with high-affinity antibodies for IgE receptors on mast cells, down regulating their effector activity (30), and yet preserving an effective anti-parasite response.

## Conflict of Interest Statement

The authors declare that the research was conducted in the absence of any commercial or financial relationships that could be construed as a potential conflict of interest.

## References

[1] Fitzsimmons CM Falcone FH Dunne DW Helminth allergens, parasite-specific IgE, and its protective role in human immunity. Front Immunol (2014) 5:61 10.3389/fimmu.2014.00061 24592267 PMC3924148

[2] Fitzsimmons CM Dunne DW Survival of the fittest: allergology or parasitology? Trends Parasitol (2009) 25:447–51 10.1016/j.pt.2009.07.004 19744885

[3] Hagan P Blumenthal UJ Dunn D Simpson AJ Wilkins HA Human IgE, IgG4 and resistance to reinfection with *Schistosoma haematobium*. Nature (1991) 349:243–5 10.1038/349243a0 1898985

[4] Pritchard DI Quinnell RJ Walsh EA Immunity in humans to *Necator americanus*: IgE, parasite weight and fecundity. Parasite Immunol (1995) 17:71–5 10.1111/j.1365-3024.1995.tb00968.x 7761110

[5] Bethony J Loukas A Smout M Brooker S Mendez S Plieskatt J Antibodies against a secreted protein from hookworm larvae reduce the intensity of hookworm infection in humans and vaccinated laboratory animals. FASEB J (2005) 19:1743–5 16037096 10.1096/fj.05-3936fje

[6] Pinot de Moira A Fulford AJ Kabatereine NB Ouma JH Booth M Dunne DW Analysis of complex patterns of human exposure and immunity to *Schistosomiasis mansoni*: the influence of age, sex, ethnicity and IgE. PLoS Negl Trop Dis (2010) 4:e820 10.1371/journal.pntd.0000820 20856909 PMC2939029

[7] Allen JE Maizels RM Diversity and dialogue in immunity to helminths. Nat Rev Immunol (2011) 11:375–88 10.1038/nri2992 21610741

[8] Pinot de Moira A Jones FM Wilson S Tukahebwa E Fitzsimmons CM Mwatha JK Effects of treatment on IgE responses against parasite allergen-like proteins and immunity to reinfection in childhood schistosome and hookworm coinfections. Infect Immun (2013) 81:23–32 10.1128/IAI.00748-12 23071136 PMC3536155

[9] Del Prete G Human Th1 and Th2 lymphocytes: their role in the pathophysiology of atopy. Allergy (1992) 47:450–5 10.1111/j.1398-9995.1992.tb00662.x 1485646

[10] Palm NW Rosenstein RK Yu S Schenten DD Florsheim E Medzhitov R Bee venom phospholipase A2 induces a primary type 2 response that is dependent on the receptor ST2 and confers protective immunity. Immunity (2013) 39:976–85 10.1016/j.immuni.2013.10.006 24210353 PMC3852615

[11] Thomas WR Smith WA Hales BJ The allergenic specificities of the house dust mite. Chang Gung Med J (2004) 27:563–9 15553602

[12] Weghofer M Dall’Antonia Y Grote M Stöcklinger A Kneidinger M Balic N Characterization of Der p 21, a new important allergen derived from the gut of house dust mites. Allergy (2008) 63:758–67 10.1111/j.1398-9995.2008.01647.x 18445190

[13] Shakib F Ghaemmaghami AM Sewell HF The molecular basis of allergenicity. Trends Immunol (2008) 29:633–42 10.1016/j.it.2008.08.007 18951844

[14] Machado DC Horton D Harrop R Peachell PT Helm BA Potential allergens stimulate the release of mediators of the allergic response from cells of mast cell lineage in the absence of sensitization with antigen-specific IgE. Eur J Immunol (1996) 26:2972–80 10.1002/eji.1830261224 8977293

[15] Woolcock AJ Peat JK Evidence for the increase in asthma worldwide. Ciba Found Symp (1997) 206:122–34 9257009 10.1002/9780470515334.ch8

[16] Upton MN McConnachie A McSharry C Hart CL Smith GD Gillis CR Intergenerational 20 year trends in the prevalence of asthma and hay fever in adults: the Midspan family study surveys of parents and offspring. Br Med J (2000) 321:88–92 10.1136/bmj.321.7253.88 10884260 PMC27429

[17] Davis MM T cell receptor gene diversity and selection. Annu Rev Biochem (1990) 59:475–96 10.1146/annurev.bi.59.070190.002355 2197981

[18] Cabaniols JP Fazilleau N Casrouge A Kourilsky P Kanellopoulos JM Most alpha/beta T cell receptor diversity is due to terminal deoxynucleotidyl transferase. J Exp Med (2001) 194:1385–90 10.1084/jem.194.9 11696602 PMC2195970

[19] Benedict CL Gilfillan S Thai TH Kearney JF Terminal deoxynucleotidyl transferase and repertoire development. Immunol Rev (2000) 175:150–7 10.1111/j.1600-065X.2000.imr017518.x 10933600

[20] Rose S Lichtenheld M Foote MR Adkins B Murine neonatal CD4+ cells are poised for rapid Th2 effector-like function. J Immunol (2007) 178:2667–78 10.4049/jimmunol.178.5.2667 17312108 PMC2112939

[21] Hebel K Weinert S Kuropka B Knolle J Kosak B Jorch G CD4+ T cells from human neonates and infants are poised spontaneously to run a nonclassical IL-4 program. J Immunol (2014) 192:5160–70 10.4049/jimmunol.1302539 24778440

[22] Inman CF Laycock GM Mitchard L Harley R Warwick J Burt R Neonatal colonisation expands a specific intestinal antigen-presenting cell subset prior to CD4 T-cell expansion, without altering T-cell repertoire. PLoS One (2012) 7:e33707 10.1371/journal.pone.0033707 22442714 PMC3307746

[23] Lindner C Wahl B Föhse L Suerbaum S Macpherson AJ Prinz I Age, microbiota, and T cells shape diverse individual IgA repertoires in the intestine. J Exp Med (2012) 209:365–77 10.1084/jem.20111980 22249449 PMC3280880

[24] Profet M The function of allergy: immunological defense against toxins. Q Rev Biol (1991) 66:23–62 10.1086/417049 2052671

[25] Palm NW Rosenstein RK Medzhitov R Allergic host defences. Nature (2012) 484:465–72 10.1038/nature11047 22538607 PMC3596087

[26] Marichal T Starkl P Reber LL Kalesnikoff J Oettgen HC Tsai M A beneficial role for immunoglobulin E in host defense against honeybee venom. Immunity (2013) 39:963–75 10.1016/j.immuni.2013.10.005 24210352 PMC4164235

[27] Wang Y Jackson KJ Chen Z Gaëta BA Siba PM Pomat W IgE sequences in individuals living in an area of endemic parasitism show little mutational evidence of antigen selection. Scand J Immunol (2011) 73:496–504 10.1111/j.1365-3083.2011.02525.x 21284686

[28] Kerzel S Rogosch T Struecker B Maier RF Zemlin M IgE transcripts in the circulation of allergic children reflect a classical antigen-driven B cell response and not a superantigen-like activation. J Immunol (2010) 185(4):2253–60 10.4049/jimmunol.0902942 20660349

[29] McSorley HJ Maizels RM Helminth infections and host immune regulation. Clin Microbiol Rev (2012) 25:585–608 10.1128/CMR.05040-11 23034321 PMC3485755

[30] Suzuki R Leach S Liu W Ralston E Scheffel J Zhang W Molecular editing of cellular responses by the high-affinity receptor for IgE. Science (2014) 343:1021–5 10.1126/science.1246976 24505132 PMC4188507

